# Comparative transcriptome and metabolome profiling reveal molecular mechanisms underlying *OsDRAP1*-mediated salt tolerance in rice

**DOI:** 10.1038/s41598-021-84638-3

**Published:** 2021-03-04

**Authors:** Yinxiao Wang, Liyu Huang, Fengping Du, Juan Wang, Xiuqin Zhao, Zhikang Li, Wensheng Wang, Jianlong Xu, Binying Fu

**Affiliations:** 1 Institute of Crop Sciences/National Key Facility for Crop Gene Resources and Genetic Improvement, Chinese Academy of Agricultural Sciences, South Zhong-Guan-Cun Street 12#, Beijing, 100081 China; 2 School of Agriculture, Yunnan University, Kunming, Yunnan China; 3 School of Agronomy, Anhui Agricultural University, Hefei, China; 4 Research Center for Perennial Rice Engineering and Technology of Yunnan, School of Agriculture, Yunnan University, Kunming, 650091 Yunnan China

**Keywords:** Gene expression analysis, Biotechnology, Molecular biology

## Abstract

Integration of transcriptomics and metabolomics data can provide detailed information for better understanding the molecular mechanisms underlying salt tolerance in rice. In the present study, we report a comprehensive analysis of the transcriptome and metabolome of rice overexpressing the *OsDRAP1* gene, which encodes an ERF transcription factor and was previously identified to be conferring drought tolerance. Phenotypic analysis showed that *OsDRAP1* overexpression (OE) improved salt tolerance by increasing the survival rate under salt stress. *OsDRAP1* affected the physiological indices such as superoxide dismutase (SOD), catalase (CAT) and malondialdehyde (MDA) to enhance redox homeostasis and membrane stability in response to salt stress. Higher basal expression of *OsDRAP1* resulted in differential expression of genes that potentially function in intrinsic salt tolerance. A core set of genes with distinct functions in transcriptional regulation, organelle gene expression and ion transport were substantially up-regulated in the OE line in response to salt stress, implying their important role in *OsDRAP1*-mediated salt tolerance. Correspondingly, metabolome profiling detected a number of differentially metabolites in the OE line relative to the wild type under salt stress. These metabolites, including amino acids (proline, valine), organic acids (glyceric acid, phosphoenolpyruvic acid and ascorbic acid) and many secondary metabolites, accumulated to higher levels in the OE line, demonstrating their role in salt tolerance. Integration of transcriptome and metabolome analysis highlights the crucial role of amino acids and carbohydrate metabolism pathways in *OsDRAP1*-mediated salt tolerance.

## Introduction

Salt, which has adverse effects on germination, plant vigor and yield, is one of the most serious environment stresses limiting crop productivity^1^. There is an urgent need to dissect the molecular mechanisms of crop salt tolerance and identify the underlying genes so that we can develop crop varieties with salt tolerance using molecular breeding and gene editing strategies.

Rice is a glycophyte and is highly sensitive to salt stress^2^. The level of salt tolerance differs between genotypes and developmental stages. Rice is more sensitive to salt at the seedling stage but moderately tolerant at the tillering stage^3,4^, and a few genotypes have been identified to be salt tolerant^5^. Salt stress activates a series of protective responses including morphological, physiological, cellular and molecular changes^2^. Improving salt tolerance to ensure rice production will require knowledge of the diverse biological processes underlying salinity tolerance.

Salt stress tolerance is a complex agronomic trait regulated by multiple genes. To date, many quantitative trait loci (QTLs) for salt tolerance in rice have been identified using bi-parental mapping populations or by genome-wide association study (GWAS). For example, Koyama et al. mapped 11 QTLs governing sodium uptake, potassium uptake, and Na^+^/K^+^ selectivity^6^. By using a population derived from a cross between two genotypes with contrasting salt tolerances, Lin et al. identified two major QTLs, namely *qSNC-7* for shoot Na^+^ concentration and *qSKC-1* for shoot K^+^ concentration^7^. A major QTL, *Saltol*, involved in shoot K^+^/Na^+^ balance was characterized in the salt tolerant variety Pokkali^8^. With the development of high-throughput genotyping by whole-genome resequencing, GWAS has become widely used for genome-wide explorations of genetic loci controlling salt tolerance^9–12^.

A number of candidate genes underlying salt tolerance in rice have been identified using functional genomics platforms. Several protein-coding genes have been found to be involved in salt stress tolerance, these genes include transcription factors (TFs) and effectors^2^. A few TFs have been identified as positive regulators of salt tolerance in rice. *SNAC1*, which encodes a NAM, ATAF, and CUC (NAC) TF, enhances salt tolerance by regulating stomatal movement^13^. *ONAC106* functions in salt tolerance as well as leaf senescence and tiller angle determination by modulating the expression of target genes that function in each respective signaling pathway^14^. *OsMYB2*, which encodes a stress-responsive MYB TF, plays a positive role in the tolerance to salt as well as cold and dehydration stress^15^. Overexpressing these TFs in transgenic rice plants could be an efficient way to improve salt stress tolerance.

Metabolites are the final output of plant responses to genetic or environmental signals^16^. Metabolome analysis revealed that many metabolites produced in plants exposed to salt stress are involved in osmotic adjustment and osmoprotection^17^. It was shown that many products of sugar and nitrogen metabolism including sucrose, fructose, glucose; amino acids and their derivatives were highly regulated in rice under long-term mild salt stress^18^. The comparative metabolome profiling found that the metabolites of diverse rice varieties were adjusted in a temporal, tissue-specific and genotype-dependent manner under high salt stress^19^. Many studies have reported individual metabolites that are involved in salt stress tolerance; for example, levels of intermediates of the tri-carboxylic acid cycle and nitrogenous components fluctuated remarkably in rice in response to salt stress^20–22^.

Integrated analysis of the transcriptome and metabolome could provide information about the relationships between genes and metabolites and the underlying complex molecular networks^23^. In our previous work, we have characterized an ERF transcription factor gene *OsDRAP1* (Drought Responsive AP2/EREBP gene) conferring drought tolerance^24^. In this study, we compared transcriptome sequencing and metabolome profiling data of *OsDRAP1*-overexpressing transgenic and wild-type plants under salt stress condition to elucidate the molecules and pathways associated with *OsDRAP1*-mediated salt tolerance.

## Results

### Phenotype differences between OsDRAP1 OE transgenic and WT lines under salt stress conditions

The *OsDRAP1* gene (LOC_Os08g31580) is 1651 bp in length with a 497 bp 5′UTR, a 311 bp 3′UTR, and a 843 bp coding region composed of one exon. The *OsDRAP1* gene encodes a polypeptide of 280 amino acids with an AP2 domain (103–166 aa) and the transactivation activity is located primarily in the region of 207–280 aa^24^.

**Figure 1:**
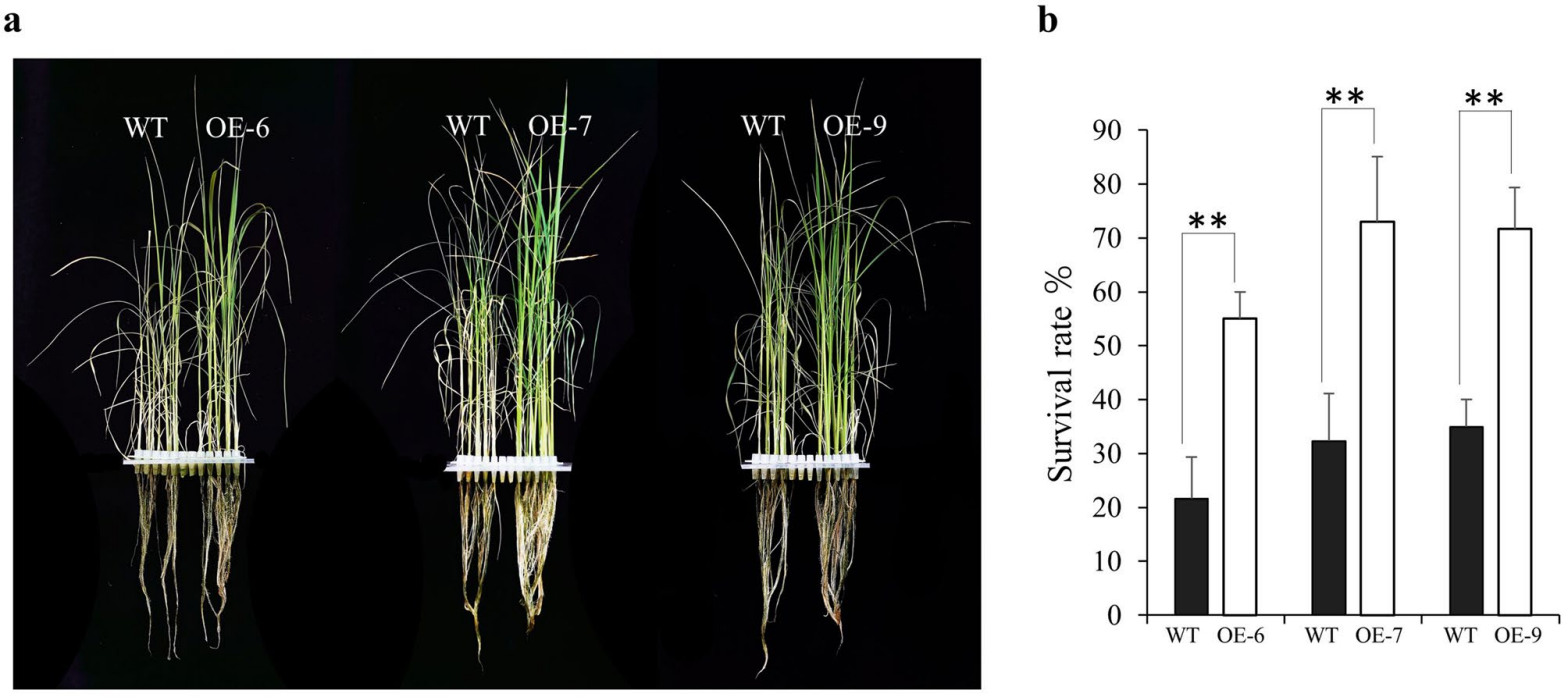
The phenotypes of *OsDRAP1* overexpression (OE) lines at the seedling stage under salt stress (120 mM NaCl). (**a**) Phenotypes of OE and WT plants after 7 days under 120 mM NaCl. (**b**) Survival rate of OE lines and WT plants after 7 days of recovery from salt stress. The asterisk indicates a significant difference determined by Student’s *t*-test (*p* < 0.05, n > 100 with three replicates).

To investigate the molecular function of *OsDRAP1* in response to salt stress, rice lines overexpressing *OsDRAP1* under UBI promoter were generated as described in our previous study^24^. Sucrose phosphate synthase gene (*OsSPS*, LOC_Os11g12810) as the reference gene and hygromycin resistance gene (*HPT*) in plasmid pCUbi1390 was used as a marker gene for quantifying transgene copy numbers analysis by qRT-PCR (quantitative Real Time Polymerase Chain Reaction)^25^. The correlation coefficients R^2^ are 0.991 and 0.979 respectively, indicating that the data is reliable (Supplementary Fig. 1a). The copy number of transgenic lines (T0 generation) was analyzed by using the equation: N × 10^(−0.303Ct1+12.397)^ = 10^(−0.368Ct2+12.806)^ (Supplementary Table S1). Three homozygous *OsDRAP1*-overexpressing lines (T3 generation) with one copy number were obtained with the criterion that all the selected 12 plants from each OE lines were positive by PCR (Polymerase Chain Reaction) validation (Supplementary Fig. 1b). The qRT-PCR results showed that the expression of *OsDRAP1* was up-regulated in three OE lines to various extents compared with wild type (WT) under the normal growth conditions (Supplementary Fig. 1c). Three OE lines (OE-6, OE-7, OE-9) were evaluated for phenotype performance under salt stress imposed by exposing three-leaf-stage seedlings of the OE lines and WT to 120 mM NaCl. After 7 days, three transgenic lines all exhibited enhanced salt tolerance relative to WT with significantly higher seedling survival rates (Fig. 1). Besides, the survival rates of OE lines were also significantly higher than WT under 150 mM NaCl treatment (Supplementary Fig. 2). These results indicated that overexpression of *OsDRAP1* improved salt tolerance in rice.

### Physiological indices of transgenic and WT lines under normal and salt stress conditions

**Figure 2:**
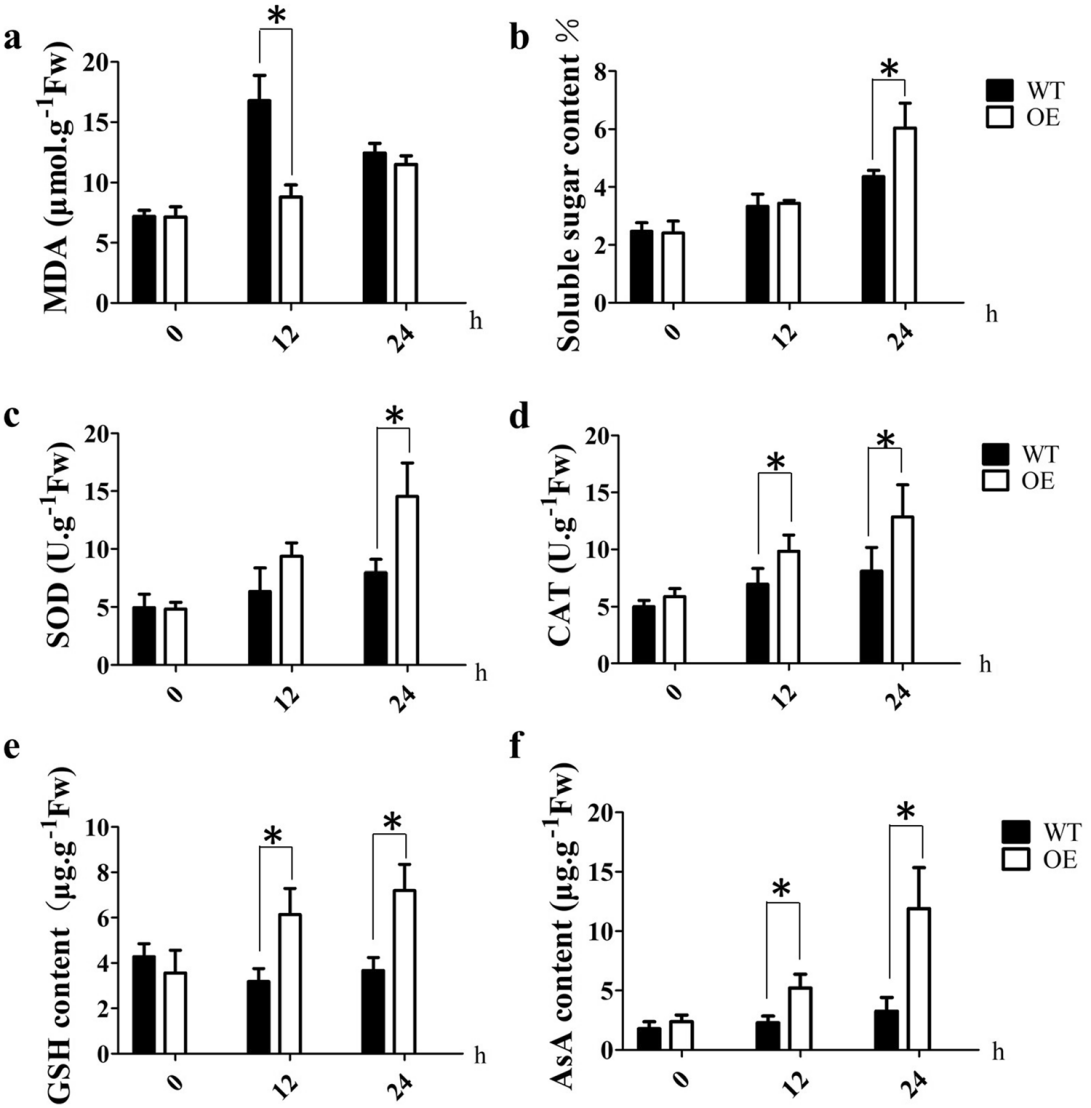
Physiological indices of the *OsDRAP1* transgenic plants (OE-7) and wild type (WT) after 0 h, 12 h and 24 h of salt stress treatment. (**a**) Malondialdehyde (MDA) content. (**b**) Soluble sugar content. (**c**) Superoxide dismutase (SOD) activity. (**d**) Catalase (CAT) activity. (**e**) Glutathione (GSH) content. (**f**) Ascorbic acid (AsA) content. The asterisk indicates a significant difference determined by Student’s *t*-test (*p* < 0.05 with three replicates).

To determine how *OsDRAP1* affects the physiological indices in response to salt stress, we comparatively analyzed the contents of malondialdehyde (MDA), soluble sugar, glutathione (GSH), and ascorbic acid (AsA), the activities of superoxide dismutase (SOD) and catalase (CAT) in OE-7 and WT plants under normal and salt stress conditions. The MDA content in the OE-7 line was significantly lower than that of WT after 12 h of salt stress, even though there was no remarkable difference between the OE and WT lines after 24 h of salt stress (Fig. 2a), indicating that the membrane system may be more stable in OE-7 than that in WT at early stages of salt stress. The soluble sugar content was significantly higher in OE-7 than in WT after 24 h of salt stress treatment (Fig. 2b). The activities of SOD and CAT, the levels of GSH and AsA were also significantly higher in OE-7 relative to WT under salt stress (Fig. 2c,d,e,f), indicating that the reactive oxygen species (ROS) scavenging ability was remarkably improved in the OE-7 line in response to salt. All these results suggested that *OsDRAP1* overexpression improved salt tolerance in transgenic rice plants by maintaining membrane integrity and enhancing the ROS-scavenging system.

### Transcriptome analysis of the OE and WT lines under normal growth condition

The transcriptomes of the OE-7 and WT lines under normal and salt stress conditions were analyzed using the RNA-seq platform. Firstly, we comparatively analyzed the global transcript levels in the OE line and WT under normal growth condition. Genes with more than two-fold change in expression in the OE line relative to WT with False discovery rate (FDR) < 0.01 were defined as the differentially expressed genes (DEGs); a total of 293 and 163 genes were found to be significantly up- and down-regulated, respectively (Supplementary Table 2).

Gene Ontology (GO) enrichment analysis revealed that the up-regulated genes were enriched in the protein modification process (GO:0006464), programmed cell death (GO:0012501) and response to stress (GO:0006950) terms (Supplementary Table 3). GO enrichment analysis showed that the down-regulated genes (Supplementary Table 4) were related to photosynthesis (GO:0015979) and generation of precursor metabolites and energy (GO:0006091), indicating that photosynthesis and energy metabolism are repressed in the OE transgenic plants.

Of the up-regulated genes, six genes encoded wall-associated receptor kinases (WAKs) (*OsWAK34*, *OsWAK60*, *OsWAK38*, *OsWAK76*, *OsWAK40* and *OsWAK59*) and several genes encoded TFs such as *OsDREB1A*, *OsDREB1B*, *OsBIERF3*, *OsERF105*, *OsNAC3*, *OsWRKY62*, *OsWRKY67* and *OsWRKY69*. All these genes up-regulated in the OE-7 line may be involved in *OsDRAP1*-mediated intrinsic salt tolerance.

To investigate common sequence motifs in the genes up-regulated in the OE-7 line relative to WT under normal condition, we identified the cis-regulatory elements in the 2-kb regions upstream of the 293 up-regulated genes. There were several highly abundant elements such as the ATCTA, CBFHV (RYCGAC), DRECRTCOREAT, and GCCCORE motifs (Supplementary Table 5). A total of 85.0% the genes contain 1–12 copies of the ATCTA motif, which has been identified as the ethylene responsive factor (ERF) binding element^26^, and around 75.4% of genes harbor 1–12 copies of the CBFHV motif, which is a dehydration-responsive element (DRE)^27^. More than 60% of the genes have at least one copy of the GCCCORE or DRECRTCOREAT motif; the GCCCORE element has an important role in regulating jasmonate-responsive gene expression^28^, and DRECRTCOREAT is a cis-element known to be recognized by AP2/ERF proteins^29^. All the enriched cis-elements could be involved in *OsDRAP1*-mediated regulation of downstream gene expression in response to salt stress.

### Comparative analysis of the transcriptomes of the OE-7 and WT under salt stress condition

A total of 825 and 1,009 genes were significantly up- and down-regulated, respectively, in OE-7 compared with WT after 24 h of salt stress. GO enrichment analysis showed that all the up-regulated genes (Supplementary Table 6) were functionally enriched in regulation of transcription (GO:0006355), post-translational protein modification (GO:0043687), photosynthesis (GO:0009765), cell wall macromolecule catabolic process (GO:0016998), oxidoreductase activity (GO:0016491), and peptide transport (GO:0015833) (Supplementary Table 7).

Among the up-regulated genes, eleven genes encoding ERF transcription factors including *OsERF2*, *OsERF3*, *OsERF61* and *OsERF62*, and other TFs such as three bZIPs (*OsbZIP09*, *OsbZIP23* and *OsbZIP62*), three MYBs (*OsMYB2*, *OsMYB30* and *OsPHR3*) and four NACs (*OMTN1*, *OsNAC6*, *OsNAC10*, *OsNAC31*) were highly induced in the OE line (Supplementary Table 6), suggesting that these TFs are involved in the *OsDRAP1*-mediated regulation of transcription in response to salt stress.

Two sodium exchanger genes, *OsCAX1b* and *OsNHX2*, a set of 87 genes encoding pentatricopeptide repeat (PPR) domain proteins and nine genes encoding proton-dependent oligopeptide transporter (POT) proteins were found to be significantly up-regulated in the OE line. These two classes (PRR and POT) of genes are reported to be involved in a wide range of biological functions^30–32^. Six chitinase genes and three genes encoding mitochondrial transcription termination factors (mTERFs) were also remarkably induced in the OE line; chitinases and mTERFs are extensively involved in biotic and abiotic stress response^33,34^. All these results suggest that over-expressing the *OsDRAP1* gene affected many TFs and genes related to stress response under salt stress conditions.

21 genes were randomly selected to verify the accuracy of the transcriptome data with qRT-PCR. The primers for qRT-PCR were listed in Supplementary Table 8, and the date were presented in Supplementary Table 9. There was a high correlation (R^2^ = 0.96) between expression levels of 21 genes determined from qRT-PCR and transcriptome data, showing that the transcriptome data were accurate and reliable (Supplementary Fig. 3).

### Metabolite profiling of the OE and WT lines in response to salt stress

**Figure 3:**
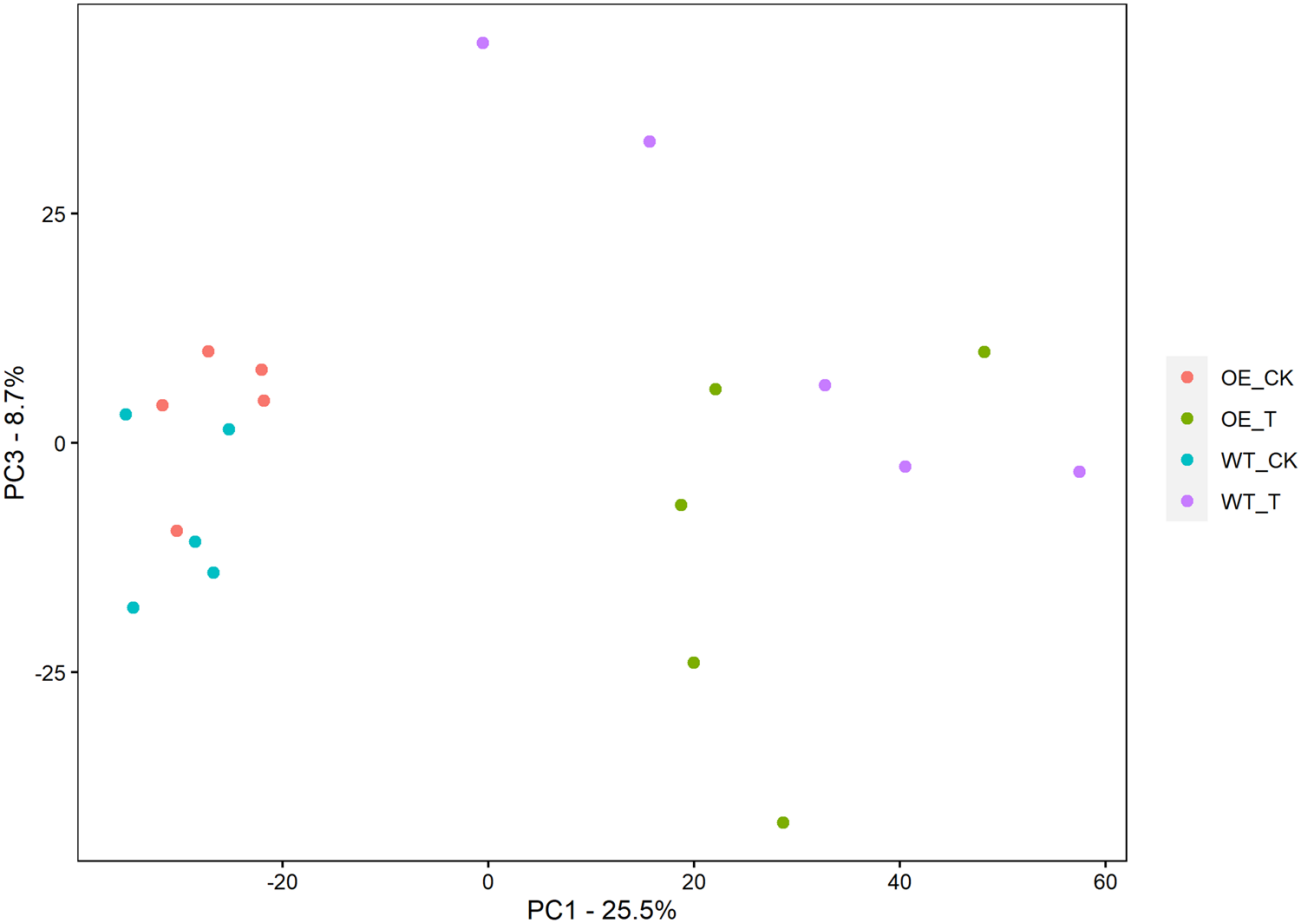
Principal component analysis (PCA) of metabolome profiling of the *OsDRAP1* transgenic plants (OE-7) and wild type (WT) with five replicates under normal growth condition and salt stress treatment for 24 h. “CK” and “T” represent the plants under normal condition and salt stress, respectively.

To globally profile the metabolite contents in leaves of the OE-7 line and WT, we performed LC/MS metabolome analysis and detected a total of 3894 biochemicals, of which 372 were known metabolites, in the two lines under normal and salt stress conditions. The metabolites covered several categories such as amino acids, carbohydrates, nucleotides, peptides, hormones and secondary metabolites (Supplementary Table 10). To reduce the dimensionality of the data and visualize the relationship among samples, we performed PCA, and the first principal component (PC1) explained 25.5% of the total variation, while the second principal component (PC3) explained 8.7% of the variation across the data set (Fig. 3). A plot of the PC1 and PC3 scores showed a clear separation in PC1 between the different treatments. The OE-7 samples and WT samples were separated by PC3 under salt stress. Compared with that under normal condition, the differences of metabolites between OE and WT samples were further increased under salt stress. This indicates changes in the metabolite profiles caused by the salt treatment as well as by the differences between the OE-7 and WT in response to salt stress.

We further performed a comparative analysis to identify the differential metabolites between the OE and WT lines as described in the Materials and Methods. Under normal growth conditions, there were 38 and 3 metabolites detected to be up- and down-regulated, respectively, in the OE line relative to WT (Supplementary Table 11). The three down-regulated metabolites were 5-amino-6-(5′-phosphoribosylamino) uracil, akuammine and paucine, and the 38 up-regulated metabolites consisted of 22 unmapped metabolites and 16 secondary metabolites including eseramine, glycerylphosphorylethanolamine, pyridaben, satratoxin H, streptidine 6-phosphate, calactin and aminoadipic acid. These results suggested that overexpression of *OsDRAP1* could elicit significant metabolite changes, especially the accumulation of secondary metabolites, under normal growth condition.

Under salt stress condition, we detected 426 differential metabolites (267 up-regulated, 159 down-regulated) in the OE line compared with WT. Of these 267 up-regulated metabolites, only 86 were known metabolites, including amino acids (proline, valine), organic acids (glyceric acid, dehydroascorbic acid, azelaic acid, phosphoenolpyruvic acid), and many secondary metabolites (Supplementary Table 12). Amino acids such as proline and valine, which are involved in osmotic stress tolerance, are very important for plant abiotic stress tolerance^35,36^. The organic acids dehydroascorbic acid, azelaic acid, glyceric acid, and aminoadipic acid were previously reported to be highly involved in plants responding to environmental stress^37–40^.

### Correlation between the transcriptome and metabolome data

To explore the correlation between gene expression and metabolite accumulation, we assessed the integrated metabolome and transcriptome profiling based on a global analysis of the covariance structure of the data sets. We detected several correlations between the levels of metabolites and genes using Pearson correlation coefficient analysis as described in the Materials and Methods. The significantly enriched pathways (those associated with significantly accumulated metabolites) in the OE line are shown in Table 1.

**Table 1:** The enriched pathways in the OE line under salt stress identified by Pearson correlation analysis of transcript and metabolite levels.

Enriched pathways	Metabolite name	Up-regulated genes	Down-regulated genes
Glycine, serine and threonine metabolism	Glyceric acid; L-Tryptophan	4	4
Cysteine and methionine metabolism	S-Methyl-5′-thioadenosine; O-Succinyl-L-homoserine	9	5
Pentose phosphate pathway	Glyceric acid	4	2
Cyanoamino acid metabolism	L-Valine	5	4
Biosynthesis of amino acids	L-Valine; L-Proline; O-Succinyl-L-homoserine; L-Tryptophan	11	6
Arginine and proline metabolism	L-Proline	4	
Aminoacyl-tRNA biosynthesis	L-Tryptophan; L-Proline; L-Valine	2	
Zeatin biosynthesis	S-Methyl-5′-thioadenosine	2	1
Valine, leucine and isoleucine degradation	L-Valine	4	1
Glycerolipid metabolism	Glyceric acid	3	3
Glyoxylate and dicarboxylate metabolism	Glyceric acid	7	2
Tyrosine metabolism	Salidroside	4	3
Phenylalanine and tryptophan biosynthesis	L-Tryptophan; Quinic acid	2	2
Sulfur metabolism	O-Succinyl-L-homoserine; Taurine	5	3
ABC transporters	Erythritol; L-Proline; L-Valine; Taurine	1	
Pyrimidine metabolism	Thymine	7	5
Taurine and hypotaurine metabolism	Taurine	4	1

Correlations with Pearson correlation coefficient > 0.80 and a Pearson correlation coefficient *P* value < 0.05 were considered significant.

There were 17 pathways highly enriched in the OE line compared with WT under salt stress conditions (Table 1), including several amino acid metabolism pathways such as glycine, serine and threonine metabolism (8 genes), cysteine and methionine metabolism (13 genes), arginine and proline metabolism (4 genes), and tyrosine metabolism (7 genes). A few carbohydrate metabolism pathways including the pentose phosphate pathway (6 genes), glyoxylate and dicarboxylate metabolism (9 genes) and taurine and hypotaurine metabolism (5 genes) were highly enriched; and biosynthesis of amino acids was also evidently enriched (17 genes) in OE line (Supplementary Table 13, Supplementary Fig. 4).

## Discussion

In a previous study, we showed that overexpression of the gene *OsDRAP1* in transgenic rice plants enhances drought tolerance without retarding growth^24^. OsDRAP1 is a transcription factor in the AP2/ERF family, members of which are involved in development and abiotic/biotic stress signal transduction in higher plants^41^. In the present study, we combined transcriptome and metabolome analysis to dissect the physiological, gene expression and metabolites changes of *OsDRAP1*-mediated salt stress tolerance. Our result showed that overexpression of *OsDRAP1* could improve rice salt tolerance. In addition, a number of genes were either up- or down-regulated, and a few metabolites were differentially produced in a transgenic *OsDRAP1* OE line relative to WT under normal and salt stress conditions, suggesting that *OsDRAP1* might have a role in regulation of the complex molecular mechanisms of salt stress tolerance at both the transcriptional and metabolic levels.

Plants subjected to salt stress undergo a wide range of physiological changes^42^. ROS such as ^1^O_2_, H_2_O_2_ and O_2_^−^ are highly reactive molecules that accumulate to high levels under abiotic stresses including salt, water deficit and extreme temperature conditions and lead to oxidative damage in plant cells^43^. ROS scavenging enzymes and antioxidants such as GSH and AsA play an important role in reducing oxidative stress^44,45^. In the present study, the activities of ROS enzymes (SOD and CAT) and the contents of GSH and AsA were significantly higher in the *OsDRAP1* OE line than in WT under salt stress condition (Fig. 2), indicating that overexpression of *OsDRAP1* could strongly enhance the ability of rice plants to maintain redox balance and protect against oxidative stress under salt stress. Meanwhile, the increased soluble sugar and decreased MDA contents in the OE line under salt stress demonstrated that *OsDRAP1* is involved in maintaining cell membrane integrity and energy supply under salt stress; a higher concentration of soluble sugars can provide carbohydrate energy in plants under various unfavorable environmental conditions^46^ and MDA is an indicator of oxidative damage in the plant cell membrane induced by stress^47,48^.

To explore the downstream genes regulated by overexpression of *OsDRAP1*, we first compared the whole genome gene expression profiles in the OE line and WT under normal growth condition. The expression of a set of genes with distinct functions was found to be up-regulated by overexpression of *OsDRAP1* (Supplementary Table 2). These genes include six WAK family genes (*OsWAK34*, *OsWAK60*, *OsWAK38*, *OsWAK76*, *OsWAK40* and *OsWAK59*), which are involved in cell wall, signaling transduction and environmental stress response^49–51^. In particular, a few TFs functioning in transcriptional regulation in response to environmental stresses were synergistically up-regulated in the OE line. For example *OsDREB1A* and *OsDREB1B* were previously characterized as cold stress regulators^29,52^; *OsBIERF3* and *OsERF105* were reported to be involved in rice development and osmotic stress tolerance^53,54^; and several WRKY genes such as *OsWRKY62*, *OsWRKY67* and *OsWRKY69* are highly involved in basal biotic stress response^55–58^. All these genes with evidently higher expression in the OE line relative to WT could play a crucial role in *OsDRAP1*-mediated stress tolerance.

Further comparative analysis of the transcriptomes after 24 h of salt stress revealed more genes differentially expressed between the OE line and WT. Several functional categories of genes including transcription regulation, photosynthesis, cell wall process, peptide transport and oxidoreductase activity were evidently up-regulated in OE line under salt stress, showing diverse functional genes are involved in salt stress response.

Fourteen TF genes including ERFs, bZIPs, MYBs and NACs had significantly higher expression in the OE line compared with WT under salt stress, implying that they coordinately regulate transcription in response to salt. For example, *OsERF61*, *OsERF63*, *OsERF2* and *OsERF3*, which are members of the same AP2/ERF subfamily, were up-regulated, and these four ERF genes were previously found to be important factors regulating growth and metabolism in plants in response to abiotic stress^59–61^. Members of a large family of NAC TFs such as *OsNAC1*, *OsNAC6*, *OsNAC10* and *ONAC131* were all up-regulated in the OE line. Overexpression of *OsNAC10* in rice roots enhances drought tolerance and grain yield^62^, and *ONAC131* plays and important role in rice disease resistance by regulating the expression of other defense- and signaling-related genes^63^. Higher expression of these TFs likely contributes to *OsDRAP1*-mediated salt stress tolerance in the OE line.

PPRs and mTERFs are involved in the regulation of organelle gene expression, and play crucial roles in plant growth and development by tightly coordinating nuclear gene expression^34,64^. Several PPR genes in rice were found to be involved in salt stress response^65^, and a few mTERFs in *Arabidopsis* were found to increase adaptability to environmental changes^66,67^. In the present study, a large set of PPR genes and three mTERF genes were up-regulated in the OE line, showing that regulation of organelle gene expression is closely associated with salt tolerance.

Eleven genes encoding POT proteins and two sodium exchanger genes (*OsCAX1b* and *OsNHX2*) were up-regulated in the OE line relative to WT, suggesting their role in osmotic regulation. POT proteins function in potassium translocation and shoot potassium homeostasis, regulating nutrient balance, growth, and stress tolerance in plants^32,68^, while *OsCAX1b* and *OsNHX2* are reported to be involved in sodium/calcium transport^69,70^. These up-regulated POT genes and sodium exchanger genes might increase the potassium content and decrease the sodium level to help maintain ion homeostasis in the OE line under stress.

The fluctuation in unique metabolites is considered essential for plant abiotic stress response; these metabolites regulate osmosis, energy balance, ROS homeostasis and signal transduction^71,72^. We first compared the metabolite contents between the OE line and WT under normal conditions and found that overexpression of *OsDRAP1* resulted in the accumulation of secondary metabolites. Aminoadipic acid, which is related to lysine metabolism, is an important signaling amino acid that regulates plant growth and responses to the environment^73^; calactin and satratoxin H are involved in program cell death, which has been widely implicated in biotic stress tolerance^74,75^; and glycerylphosphorylethanolamine is associated with osmotic stress tolerance^76^. These accumulated metabolites could have priming effect of *OsDRAP1* OE line for stress tolerance.

The results presented in this study revealed alterations in large numbers of metabolites in OE relative to WT under salt stress, consistent with the findings of a previous study^19,20^. Amino acids including proline and valine accumulated under salt stress, suggesting that these metabolic pathways are related to *OsDRAP1*-mediated salt tolerance. Levels of several organic acids such as glyceric acid, azelaic acid, phosphoenolpyruvic acid and dehydroascorbic acid were higher in the OE line under stress. Up-regulation of glyceric acid was previously found to contribute to ROS scavenging under stress^77^. Increased levels of azelaic acid, which is involved in lipid peroxidation, enhance biotic stress tolerance^78^. A higher AsA content contributes to the protection of thylakoid membrane lipids from oxidation in stressed plants^40^. All these results indicate that the accumulation of specific amino acids and organic acids plays an important role in salt tolerance of the OE line.

Further correlation analysis revealed that several metabolism pathways were specifically enriched in the *OsDRAP1* OE line, illustrating the changes in metabolism associated with salt stress response. Especially a few amino acid metabolism pathways related to salt stress were determined. Amino acids are not only indispensable for protein synthesis but also play important cellular functions. Arginine and proline have positive effects on membrane integrity along with adaptive roles in mediating osmotic adjustment in plants under stress conditions^79^. The cysteine and methionine metabolism pathway is very important for the induction of the alternative oxidase pathway in poplar response to salt stress^80^. The valine, leucine and isoleucine degradation pathways play critical roles in the regulation of energy homeostasis and nutrition metabolism in plants under environmental stress^81^, and cyanoamino acid metabolism is positively related to biotic stress tolerance^82^. Additionally, carbohydrate metabolism homeostasis plays an important role in plant stress response, and the pentose phosphate pathway is essential for the ROS scavenging activity of the ascorbate–glutathione redox cycle in response to plant stress^23^. The high levels of glyoxylate and dicarboxylate metabolites in the OE line suggests that they play roles in energy supply and protection against environmental stress, which is consistent with the findings of a previous study^83^. All these results revealed that a complex molecular mechanism is involved in salt stress tolerance in the OE line.

## Conclusion

Overexpression of *OsDRAP1* improves rice salt stress tolerance by enhancing redox homeostasis and membrane stability. Combined transcriptome and metabolome analysis revealed a complex molecular mechanism underlying *OsDRAP1*-mediated salt stress tolerance; higher expression of *OsDRAP1* caused differential expression of genes and metabolites, which could be involved in basal stress tolerance of the OE line. A number of genes with diverse functions including transcription regulation, organelle gene expression and ion transportation were expressed at higher levels in the *OsDRAP1* OE line under salt stress; metabolites such as amino acids, organic acids and secondary metabolites also accumulated to high levels in the OE line under salt stress, suggesting their important roles in salt tolerance. Combined transcriptome and metabolome analysis highlights the crucial role of amino acids and carbohydrate metabolism in *OsDRAP1*-mediated salt tolerance.

## Materials and methods

### Plant materials, growth conditions and stress treatments

To evaluate salt tolerance phenotypes, *Oryza sativa* L. cv. Nipponbare, a japonica rice cultivar, was used to generate *OsDRAP1* overexpression (OE) lines by *Agrobacterium*-mediated transformation^25^. The background parent Nipponbare as wild-type (WT) control plants and the OE lines were grown in Yoshida nutrient solution^84^ under controlled conditions with 14 h of daylight at 28 °C and 10 h of darkness at 25 °C. The formula of Yoshida nutrient solution was listed in the Supplementary Table 14. At the three-leaf seedling stage, three OE lines and WT were subjected to salt stress by transferring to nutrient solution containing 120 mM and 150 mM NaCl for 7 days, then cultured in Yoshida nutrient solution without NaCl for 7 days. The plants were photographed when the salt phenotype was observed, and the survival rate (%) was analyzed statistically using a Student’s *t*-test.

### Physiological analysis

One OE line (OE-7) and WT at the three-leaf stage were subjected to salt treatment by transferring to nutrient solution containing 120 mM NaCl for 24 h. The leaves from three plants were collected and analyzed for soluble sugar content, malondialdehyde (MDA) content, superoxide dismutase (SOD) activity, catalase (CAT) activity, and contents of antioxidants, namely glutathione (GSH) and ascorbic acid (AsA). MDA content and total soluble sugar concentrations were measured using the method previously described by Song et al.^85^, and the SOD and CAT activities were estimated as described by Ouyang et al.^86^. GSH^87^ and AsA^88^ contents were calculated as described previously. All data were analyzed using the Student's *t*-test with three replicates.

### Transcriptome analysis

Salt stress was induced by transferring plants to nutrient solution containing 120 mM NaCl as above described. The aerial parts of seedlings at the three-leaf stage were harvested at 24 h after salt treatment and normal growth condition (three replicates for each sample). Total RNA was extracted from sampled leaves by using TRIzol Reagent (Invitrogen, USA). RNA concentration was measured using a NanoDrop 2000 (Thermo Scientific). RNA integrity was assessed using the RNA Nano 6000 Assay Kit of the Agilent Bioanalyzer 2100 system (Agilent Technologies, CA, USA). RNA sequencing and assembly were performed by Biomarker Technologies Corporation (Beijing). Analysis of differential expression between two samples was performed using EBseq. False discovery rate (FDR) < 0.01, |log2 (fold change)|≥ 2 was set as the threshold for significant differential expression. Gene Ontology (GO) enrichment analysis of the differentially expressed genes (DEGs) was implemented by the GOseq R package based on the Wallenius non-central hyper-geometric distribution^89^, which can adjust for gene length bias in DEGs. The expression levels of the selected DEGs were further confirmed by qRT-PCR.

### Metabolome analysis

The materials were the same as those used for transcriptome analysis. The aerial parts of seedlings at the three-leaf stage were harvested at 24 h after salt treatment and normal growth condition (five replicates for each sample). Metabolite extraction and LC–MS/MS analysis were performed by Biomarker Technologies Corporation (Beijing). About 500 mg accurately weighed sample was transferred to a 1.5 mL Eppendorf tube. 20 μL of 2-chloro-l-phenylalanine (0.3 mg/mL) dissolved in methanol as internal standard and 1 mL mixture of methanol and water (7/3, vol/vol) were added to each sample, samples were placed at − 80 °C for 2 min. Then grinded at 60 Hz for 2 min, and ultrasonicated at ambient temperature for 30 min after vortexed, then placed at 4 °C for 10 min. Samples were centrifuged at 13,000 rpm, 4 °C for 15 min. The supernatants from each tube were collected using crystal syringes, filtered through 0.22 μm microfilters and transferred to LC vials. The vials were stored at − 80 °C until LC–MS analysis. LC–MS/MS analyses were performed using an UHPLC system (1290, Agilent Technologies) with a UPLC BEH Amide column (1.7 μm 2.1 * 100 mm, Waters) coupled to a TripleTOF 5600 (Q-TOF, AB Sciex). The acquisition software (Analyst TF 1.7, AB Sciex) continuously evaluates the full scan survey MS data. MS raw data (.d) files were converted to mzXML format using ProteoWizard, and processed using the R package XCMS (version 3.2)^90–92^.

Negative-ion ESI mode provided better sensitivity and more observable peaks in total ion chromatograms than positive-ion mode; therefore, we used negative-ion mode as the main profiling strategy, and used positive-ion mode to obtain additional information for metabolite identification as previously described^93^.

The fold change, the *P* value from Student's t test and the variable importance in the projection (VIP) value from the orthogonal projections to latent structures- discriminant analysis (OPLS-DA) model were combined to identify the differential metabolites, using a threshold of with |fold change|> 2, *P* value < 0.05 and VIP > 1.

### Principal component analysis

Principal component analysis (PCA) of metabolites was performed using the R package XCMS (version 3.2). The package prcomp was used for the analysis and factoextra was used for ggplot2-based visualization. The abscissa of the PCA score chart represents the first principal component, namely PC1, and the ordinate represents the second principal component, namely PC3.

### Metabolite-transcript correlation analysis

Pearson correlation coefficients (PCCs) were calculated for metabolite and transcript profile data as described by Cho et al.^94^ and Reem et al.^95^. The mean of all the biological replications of the OE line and WT for metabolite data and the normalized mean value of each gene for transcriptome data were evaluated. The coefficients were calculated using the log_2_ (fold change) values of each metabolite and gene. PCC > 0.80 and PCC P value (PCCP) < 0.05 were used as the criteria for screening.

### Quantitative RT-PCR analysis

Total RNA was extracted from rice leaves using Trizol (Invitrogen) and the Direct-zol RNA MiniPrep Kit (Zymo Research) according to the manufacturer’s instructions. The FastKing gDNA Dispelling RT SuperMix (TIANGEN Biotech Co., Beijing) was used for the synthesis of first-strand cDNA. qRT-PCR using the primers listed in Supplementary Table 8 was carried out with SuperReal PreMix Plus (SYBR Green) (TIANGEN Biotech Co., Beijing) following the manufacturer’s instructions. qPCR was performed with an Applied Biosystems 7500 thermocycler (Thermo Fisher Scientific, USA) under the following cycling conditions: 95 °C for 15 min, followed by 40 cycles of 95 °C for 10 s and 60 °C for 32 s. The expression of rice *Actin1* (LOC_Os03g50890) was used as an internal control to normalize target gene expression. The relative expression of each gene was calculated based on the 2^−△△CT^ method^96^.

## Supplementary Information

Supplementary Figures.

Supplementary Tables.
