# Supplementary material for: Comparative transcriptome and metabolome profiling reveal molecular mechanisms underlying OsDRAP1-mediated salt tolerance in rice
Source: Sci Rep. 2021 Mar 4;11:5166. doi: 10.1038/s41598-021-84638-3 (PMC7933422; doi:10.1038/s41598-021-84638-3)
Supplement: Supplementary file 1 — Supplementary Figures. [file 41598_2021_84638_MOESM1_ESM.pptx]

## Slide 1
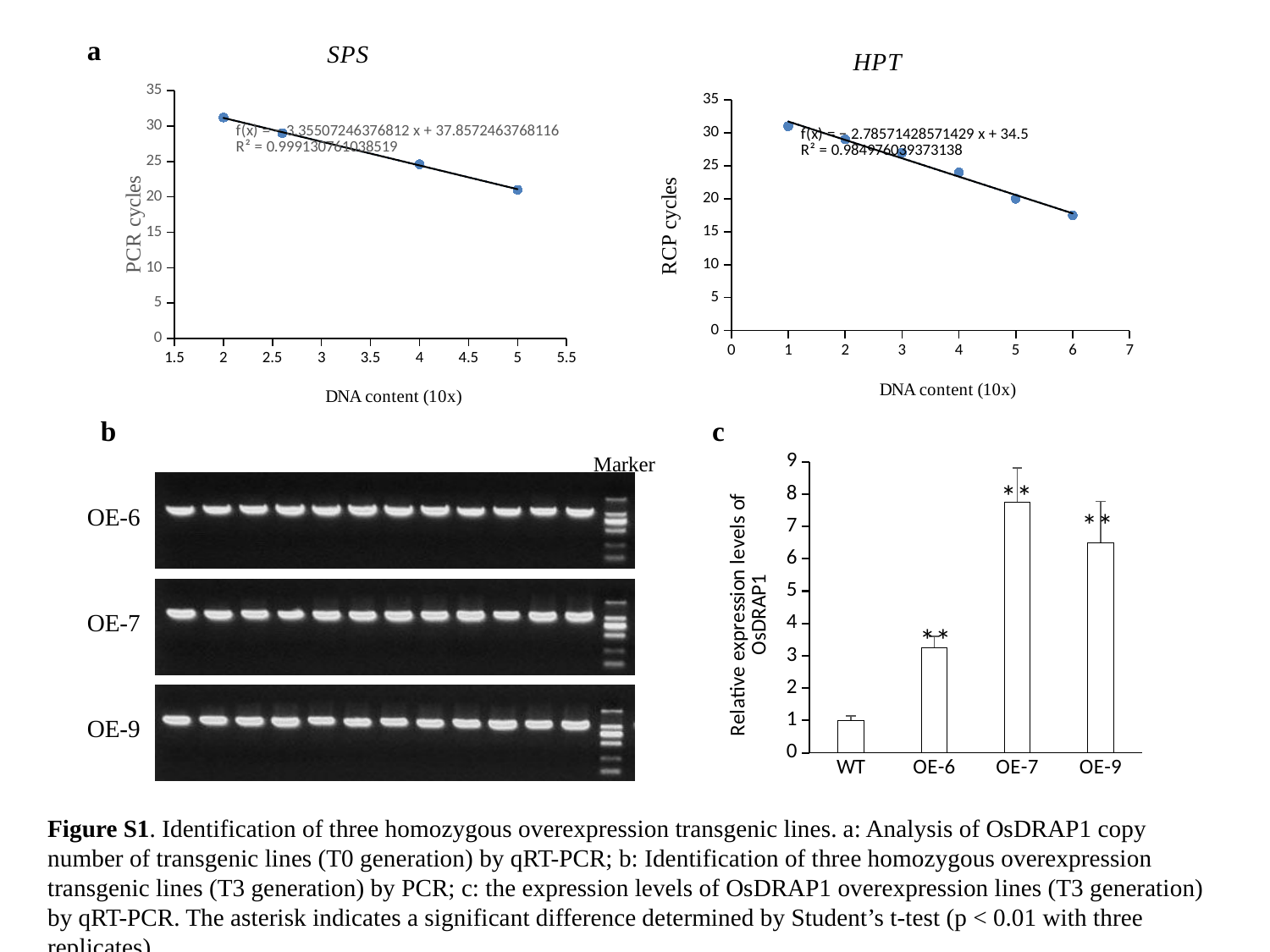

### Chart: SPS
| Category | |
|---|---|a
b
c
### Chart
| Category | |
|---|---|
| WT | 1.0 |
| OE-6 | 3.25 |
| OE-7 | 7.75 |
| OE-9 | 6.5 |**
**
**
Marker
OE-6
OE-7
OE-9
### Chart: HPT
| Category | |
|---|---|Figure S1. Identification of three homozygous overexpression transgenic lines. a: Analysis of OsDRAP1 copy number of transgenic lines (T0 generation) by qRT-PCR; b: Identification of three homozygous overexpression transgenic lines (T3 generation) by PCR; c: the expression levels of OsDRAP1 overexpression lines (T3 generation) by qRT-PCR. The asterisk indicates a significant difference determined by Student’s t-test (p < 0.01 with three replicates).

## Slide 2
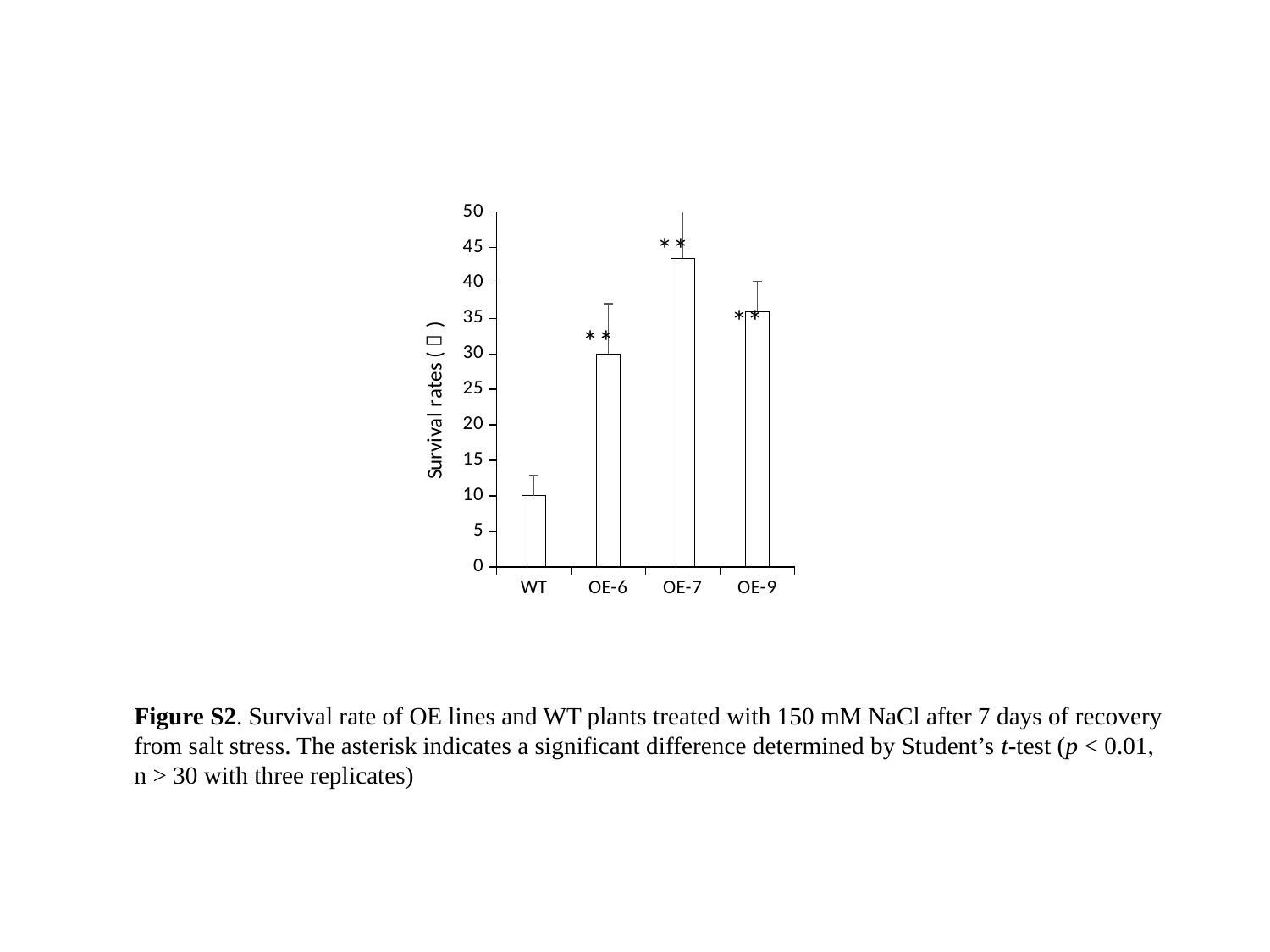

### Chart
| Category | |
|---|---|
| WT | 10.0 |
| OE-6 | 30.0 |
| OE-7 | 43.5 |
| OE-9 | 36.0 |**
**
**
Figure S2. Survival rate of OE lines and WT plants treated with 150 mM NaCl after 7 days of recovery from salt stress. The asterisk indicates a significant difference determined by Student’s t-test (p < 0.01, n > 30 with three replicates)

## Slide 3
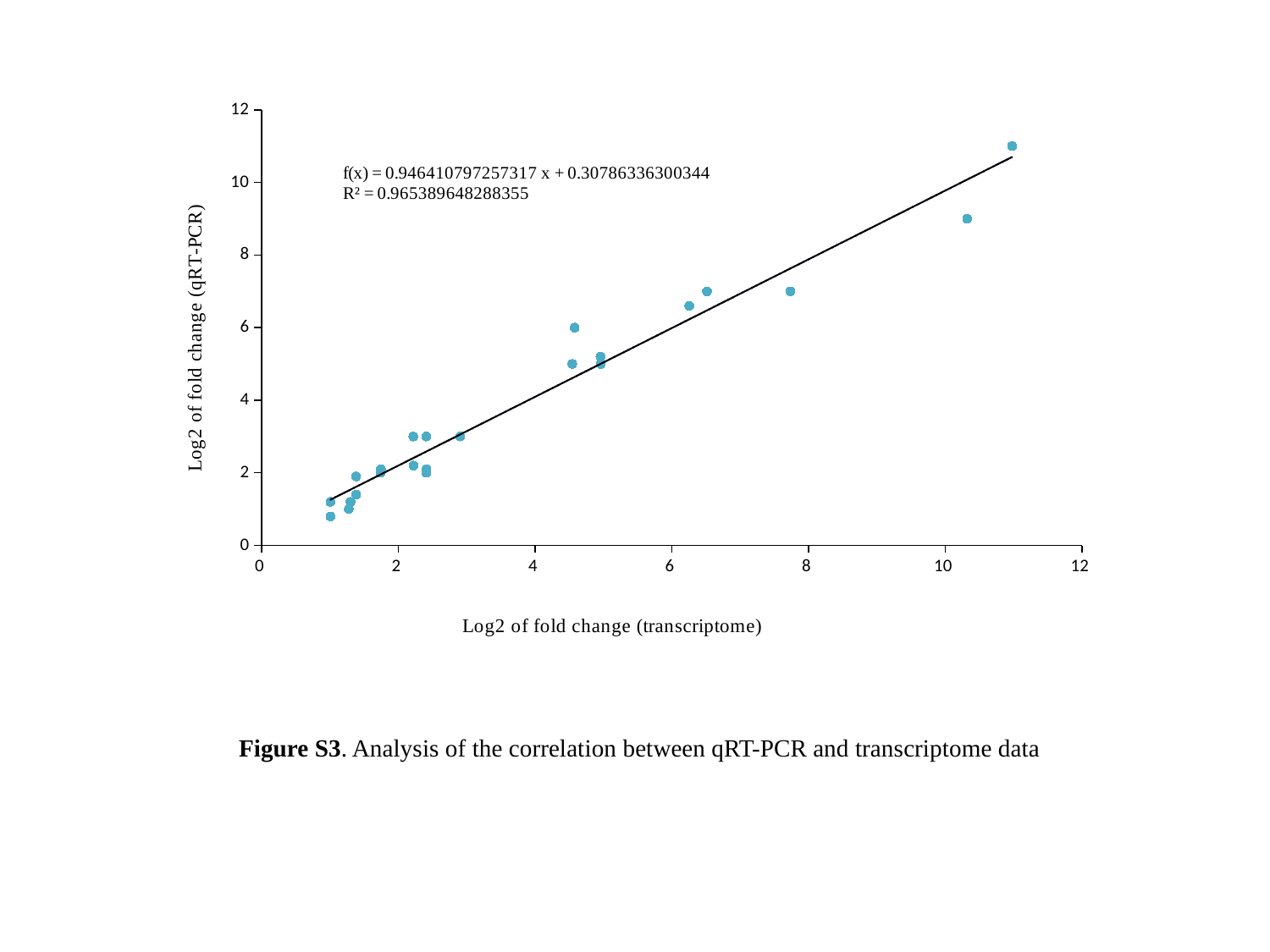

### Chart
| Category | |
|---|---|Figure S3. Analysis of the correlation between qRT-PCR and transcriptome data

## Slide 4
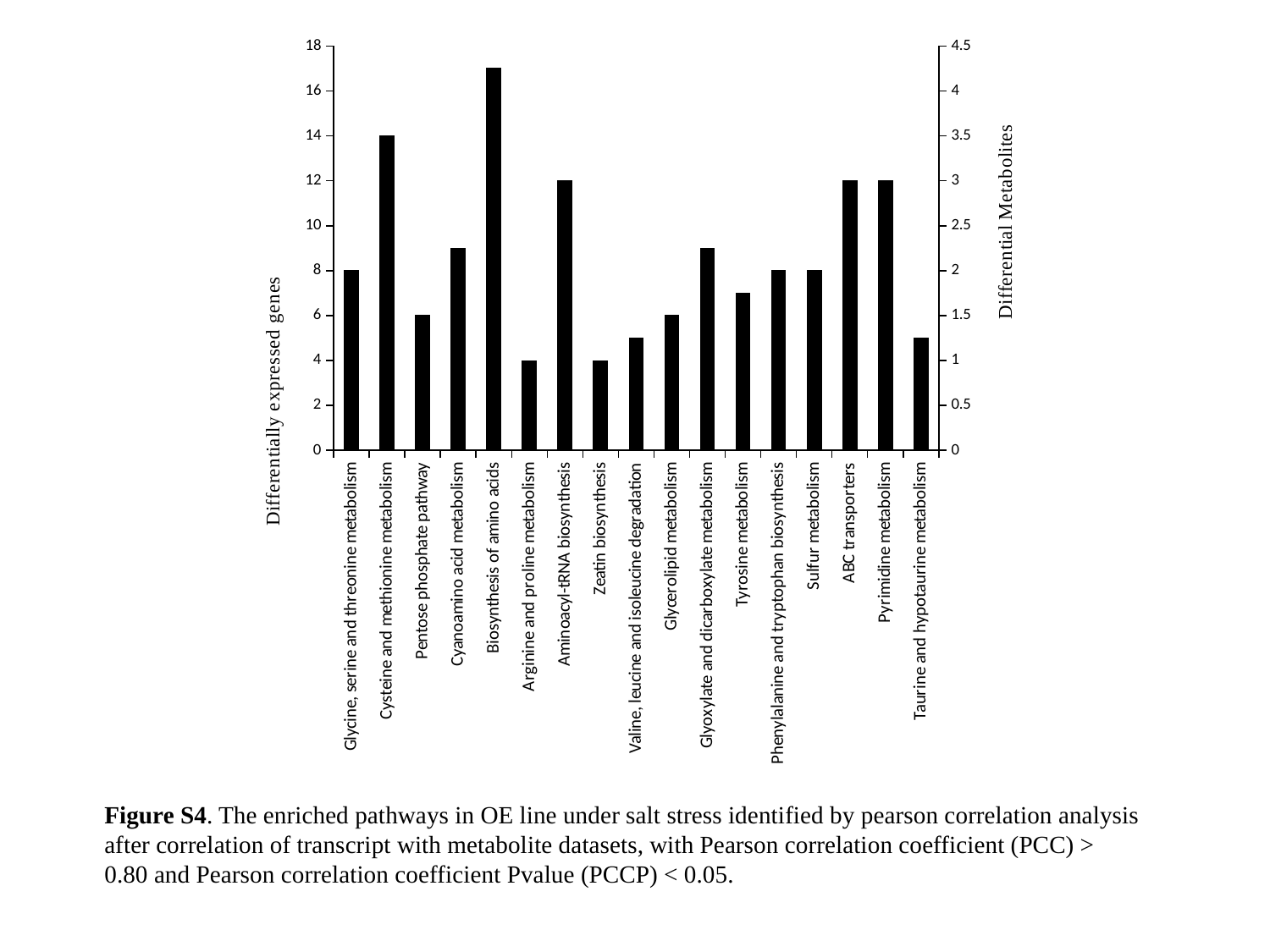

### Chart
| Category | differentially expressed genes | differential Metabolites |
|---|---|---|
| Glycine, serine and threonine metabolism | 8.0 | 2.0 |
| Cysteine and methionine metabolism | 14.0 | 2.0 |
| Pentose phosphate pathway | 6.0 | 1.0 |
| Cyanoamino acid metabolism | 9.0 | 1.0 |
| Biosynthesis of amino acids | 17.0 | 4.0 |
| Arginine and proline metabolism | 4.0 | 1.0 |
| Aminoacyl-tRNA biosynthesis | 2.0 | 3.0 |
| Zeatin biosynthesis | 3.0 | 1.0 |
| Valine, leucine and isoleucine degradation | 5.0 | 1.0 |
| Glycerolipid metabolism | 6.0 | 1.0 |
| Glyoxylate and dicarboxylate metabolism | 9.0 | 1.0 |
| Tyrosine metabolism | 7.0 | 1.0 |
| Phenylalanine and tryptophan biosynthesis | 4.0 | 2.0 |
| Sulfur metabolism | 8.0 | 2.0 |
| ABC transporters | 1.0 | 3.0 |
| Pyrimidine metabolism | 12.0 | 1.0 |
| Taurine and hypotaurine metabolism | 5.0 | 1.0 |Figure S4. The enriched pathways in OE line under salt stress identified by pearson correlation analysis after correlation of transcript with metabolite datasets, with Pearson correlation coefficient (PCC) > 0.80 and Pearson correlation coefficient Pvalue (PCCP) < 0.05.
